# *Helicoverpa zea* Boddie (Lepidoptera: Noctuidae) pupal success and adult eclosion across variable soil type and moisture

**DOI:** 10.1093/ee/nvae045

**Published:** 2024-05-23

**Authors:** Igor S Schardong, Dominic D Reisig, Taynara Possebom, Joshua Heitman

**Affiliations:** Department of Entomology and Plant Pathology, North Carolina State University, Gardner Hall 2306, 100 Derieux Pl., Raleigh, NC 27607, USA; Department of Entomology and Plant Pathology, North Carolina State University, Vernon G. James Research and Extension Center, 207 Research Station Rd., Plymouth, NC 27962, USA; Department of Entomology and Plant Pathology, North Carolina State University, Gardner Hall 2306, 100 Derieux Pl., Raleigh, NC 27607, USA; Department of Crop and Soil Sciences, North Carolina State University, Williams Hall 3410, 101 Derieux Pl., Raleigh, NC 27695, USA

**Keywords:** bollworm, corn earworm, burrowing, behavior

## Abstract

*Helicoverpa zea* Boddie (Lepidoptera: Noctuidae) is an important pest in many crops in the southern United States. Upon reaching the final larval instar, *H. zea* quests for a pupation site in the soil. Pupae are vulnerable to mortality since their movement is limited. Soil type and moisture can influence *H. zea* emergence, but the interaction of these factors has not been demonstrated. We compared sandy and clay soils in greenhouse and laboratory experiments. In the first experiment, we evaluated the preference of larvae to choose either sandy or clay soil for pupation. In a second experiment, we set the sandy soils at different moisture levels and observed prepupae pupation preference in a choice scenario. In a third experiment, we observed prepupae pupation in different moisture levels in a no-choice scenario. In a 4th experiment, we evaluated adult emergence following pupation when we increased moisture or kept it constant. In a final experiment, we evaluated pupation behavior in sandy or clay soils with a webcam and a glass arena. We found that larvae preferred to pupate in sandy soils over clay soils and that pupal success was highest at intermediate moisture levels. In addition, elevated soil moisture levels did not impact the emergence of *H. zea* between sandy or clay soil. Finally, *H. zea* did not take longer to burrow in either sandy or clay soil, but the tunnels of the pupal burrow were larger in sandy soil compared to clay soil. Our results clarify *H. zea* behavior across soil moisture and soil type.

## Introduction

The knowledge of insect behavior prior to pupation is important to understanding the ecology of those insects that pupate and their interactions with the environment. Some lepidopteran larvae quest for a place to burrow and pupate in the soil to seek protection against parasitoids, predators, and unfavorable abiotic factors (East 1974, Lindstedt et al. 2019). Soil temperatures tend to be more constant than air, and if air temperatures are lower than the lethal level for that species, a pupa in the soil might increase the odds of survival in a cold environment (Morey et al. 2012). Moreover, because the somewhat sessile pupal stage is a vulnerable phase of the insect life cycle (Lindstedt et al. 2019), the study of pupation for agricultural pests under controlled conditions might eventually lead to the implementation of Integrated Pest Management practices that disturb the soil, such as tillage or irrigation. For example, cultivation of stubble is recommended as cultural control to destroy *Helicoverpa armigera* overwintering in the soil in Australia (Fitt 1994).

*Helicoverpa zea* is an important pest of cotton and other crops in the United States (Reay-Jones 2019). Past research has investigated the impact of abiotic factors on the prepupal and pupal stages, as well as the eclosion of the pupa to the adult stage (Roach and Hopkins 1979, Dillard et al. 2023a). For example, soil type, moisture, and temperature can interact to influence *H. zea* pupal survival (Blanchard 1942, Slosser et al. 1975), and this has been shown experimentally in the field. Moreover, when sandy, clay, and high organic muck soils were infested with *H. zea* prepupae larvae, survivorship was lower, and pupae were shallower in clay soil compared to sandy soil and a high organic muck soil (Dillard et al. 2023a).

The physical properties of these soils may be important for pupal depth and survival. For example, sandy soils drain better than clay soils. This may allow prepupae larvae to burrow deeper and avoid temperature variations in sandy soils compared to clay soils. In contrast, clay soils do not drain as well as sandy soils and tend to crack when they dry. In clay soils, *H. zea* pupae are generally shallower compared to sandy soils (Dillard et al. 2023a). Although this has not been demonstrated experimentally, the increased moisture due to slow drainage in clay soils might contribute to the death of the pupae (Blanchard 1942, Parencia 1964). In addition, in-season and overwintering pupae survival has been shown to be lower in clay soil compared to sandy soil and a high organic muck soil (Dillard et al. 2023a). Therefore, sandy soils allow prepupae to burrow deeper, which may protect them from abiotic factors, increasing the survivorship of pupae compared to clay soils (Hardwick 1965).

Soil moisture and soil type may also affect adult eclosion from the pupal state and adult emergence of *H. zea*. For example, 1 experiment showed that a lack of moisture negatively influenced adult emergence in clay soils; however, the same was not observed for sandy soil and high organic muck (Dillard et al 2023b). In addition, a study in clay soil with the related *Helicoverpa punctigera* and *H. armigera*, found that when prepupae were released and simulated rainfall was applied, adult eclosion was lower in dry soil compared to wet soil (Murray and Zalucki 1990). One proposed mechanism for this phenomenon is that rainfall might disturb the prepupae burrow and pupal cells, reducing adult eclosion (Roach and Hopkins 1979). In contrast, soil type does not always influence noctuid adult eclosion. For example, in 1 study comparing sandy soil types with similar texture compositions, adult eclosion of *H. zea* and *Heliothis virences* was consistent across soil types (Roach and Hopkins 1979). As a result, it appears that significant differences in soil texture greatly impact the emergence of adult *Helicoverpa* in dry soil, emphasizing the crucial role of moisture in facilitating pupation and adult emergence. Additionally, the behavior of prepupae across soils with diverse compositions has not been studied. Such studies could potentially help explain why adult emergence is lower in dry soil conditions.

We designed a series of studies to investigate the interaction of soil type and moisture on *H. zea* prepupae larvae behavior and adult emergence to fill knowledge gaps in this area of *H. zea* ecology. We expected prepupae larvae to burrow more successfully into sandy soils compared to clay soils, to burrow deeper and longer in sandy compared to clay soils, and to prefer to burrow and more successfully complete pupation in intermediate moisture levels compared to low or high moisture levels.

## Materials and Methods

### Soil Collection, Analysis, and Preparation for Experiments

We collected 2 different soils by hand from the field in North Carolina up to a depth of 50 cm. We sent samples of both soils to the Laboratory of Soil Physical Properties at North Carolina State University to analyze particle size and water retention capacity. The soils are classified as Grossarenic Kandiudults (clay = 3.0%, silt = 7.2%, and sand = 89.8%) and Ultic Hapludalfs (clay = 26.5%, silt = 30.8%, and sand = 42.7%). Since the first soil contained a high amount of sand, and the latter contained a high amount of silt and clay, the first will be referred to as “sandy” and the second “clay.” We also sent soil samples to the North Carolina Department of Agriculture & Consumer Services Laboratory for a chemical properties analysis (Table 1). We sieved the soils with a 0.63 cm mesh metal, dried them under sunlight for 2 days, and then autoclaved for 20 min at 120 °C to prevent pathogen presence.

**Table 1.** Chemical and physical properties of sandy and clay soils

Properties	Unit	Sandy	Clay
Sand	(%)	89.8	42.7
Silt	(%)	7.2	30.75
Clay	(%)	3	26.5
pH		6.4	5.8
P	(mg dm^−3^)	153	115
K	(mg dm^−3^)	33	75
S	(mg dm^−3^)	42	80
Base SaturationV	(%)	82	77
Ca	(cmolc dm^−3^)	64	56
Mg	(cmolc dm^−3^)	13	16
Mn	(cmolc dm^−3^)	109	482
Zn	(cmolc dm^−3^)	407	291
Cu	(cmolc dm^−3^)	98	345
CEC	(cmolc dm^−3^)	3.8	7.2

Each soil naturally packed to a different soil bulk density, and thus had a different porosity under experimental conditions. For our experimental conditions, we defined soil moisture conditions based on moisture levels representing the proportion of the pore space (i.e., porosity) that was filled with water. Thus, soil volumetric water content (m^3^ m^−3^) at each condition can be considered as the product of each soil’s porosity (m^3^ m^−3^) and the corresponding experimental moisture level (%), as shown in Table 2. We also conducted water retention measurements to allow us to compare the experimental moisture levels to typical water contents at field conditions. We characterized the water retention of the soils through a ceramic pressure plate extractor analysis (Klute 1986) with 4 replicates for each soil at 33 kPa and 1.5 MPa pressures, considered representative of soil at field capacity (FC) and at permanent wilting point (PWP), respectively. For our sandy soil, water contents at FC and at PWP were 0.04 and 0.01 m^3^ m^−3^, which correspond to moisture levels of 24% and 5%, respectively. For the clay soil, FC and PWP correspond to water contents of 0.17 and 0.09 m^3^ m^−3^, and moisture levels of 54% and 29%, respectively.

**Table 2.** Soil moisture levels used based on volumetric water content related to porosity

Soil type	Treatment (moisture level %)	Porosity (m^3^/m^3^)	Volumetric water content (m^3^/m^3^)	Air-filled porosity (m^3^/m^3^)
Sand	5	0.41	0.02	0.39
	25	0.41	0.10	0.31
	50	0.41	0.21	0.20
	80	0.41	0.33	0.08
Clay	25	0.56	0.14	0.42

### Helicoverpa zea Culture and Diet Preparation

We purchased eggs of *H. zea* from Benzon Research Inc. (Carlisle, PA, USA) and fed the larvae on an artificial diet (*H. zea* diet; Southland Products, Lake Village, AR, USA). We placed each larva individually into 29.57ml plastic cups (Comfy package, Rikkel Corporation, China). We reared the larvae at 24 °C, at relative moisture of 60%, and at L:D 10:14. We observed larvae daily and selected those that reached 6th instar. To decrease contamination in the experiments, we submerged the selected larvae in a bleach solution (3.5%) or alcohol 70% followed by distilled water to sterilize and clean the insects from diet leftovers.

### Soil Type No-choice Larval Behavior Prior to Pupation Experiment

**Fig. 1.**
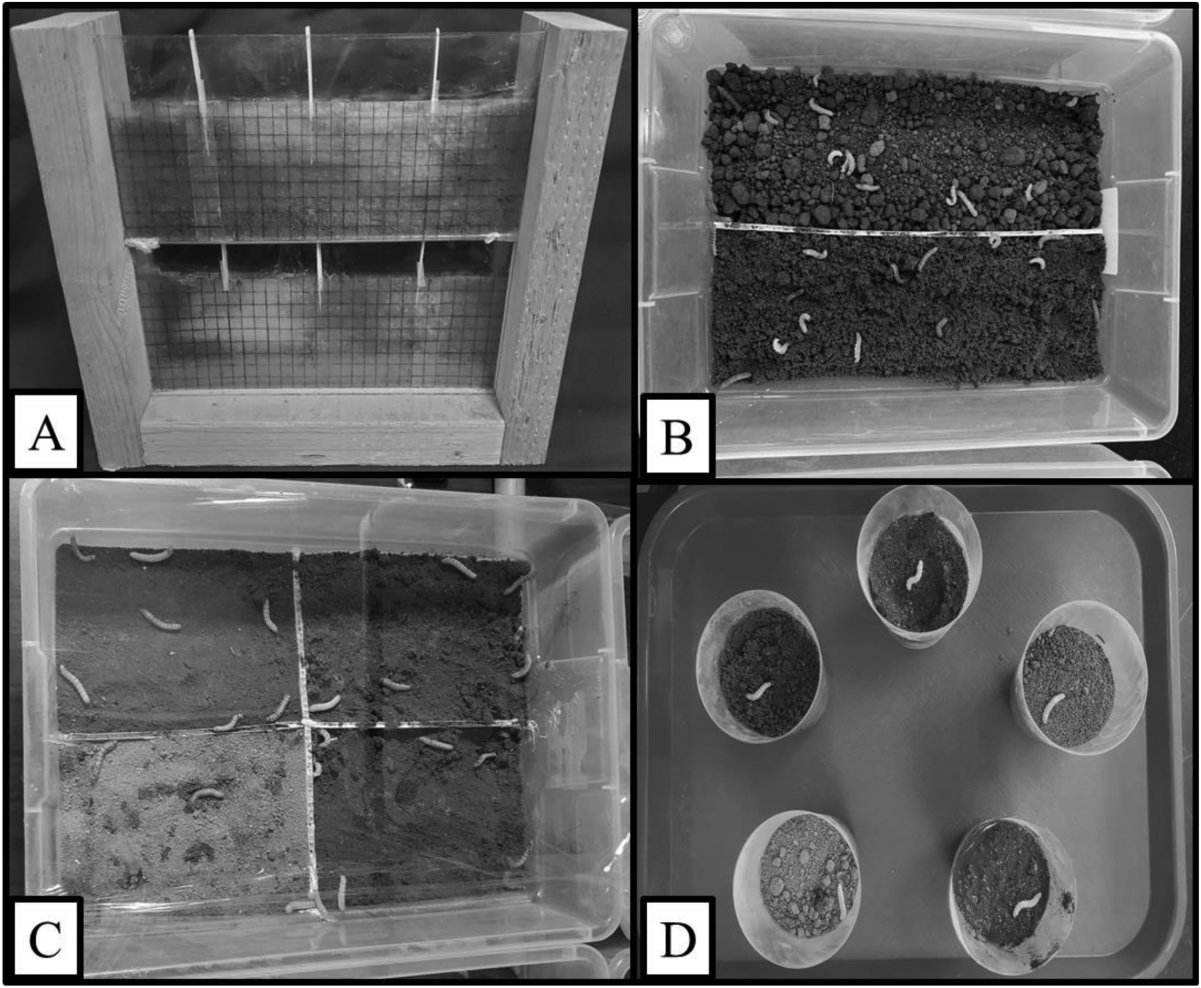
A) Arena with sandy soil used for the soil type no-choice larval behavior prior to the pupation experiment. B) Box used for the soil type choice experiment. C) Box used for the soil moisture choice experiment. D) Cups used for the soil moisture no-choice experiment pupation and adult emergence.

We placed a webcam (Model 960-000764) 76 cm away from 2 transparent glass frames set side by side and recorded the burrowing behavior of *H. zea* for both soils. We built 1 frame for each soil type. Our frame maintained the soil at a 0.5 cm width and was bounded on both sides by thin glass (0.5 cm × 20 cm × 25 cm). We further divided the frame into 8 sections, measuring approximately 10 cm × 6.5 cm per section. We filled each section with 8 cm soil, leaving 2 cm open. We then laid a transparent piece of plastic (10 cm × 25 cm) with small grid lines in front of the frame so that we could estimate the length of *H. zea* tunnels under the soil (Fig. 1). Then we added 1 prepupa *H. zea* larva into each of the 8 sections. We evaluated pupal depth, area of the tunnels [defined as the area (cm^2^) of the soil the prepupae larvae moved to tunnel and build their pupal chamber], time to initiate burrowing (defined as the time from release until the prepupae larvae began to use their mandibles to move soil for burrowing), time spent burrowing (defined as time from initiation of burrowing until they ceased movement in the pupal chamber), and time to finish pupation (defined as time from release until the insect completed metamorphosis from larvae to pupae). We standardized the moisture level for both soil types at 25% and kept the temperature constant at 24 °C. We repeated the experiment 5 times for each soil type. We released 80 total larvae in this experiment, 8 prepupae larvae for every replication in the frames with clay (*n* = 40), and 8 prepupae larvae for every replication in the frame with sand (*n* = 40). Due to issues with the recording software, only 3 replicates were available to evaluate the time to initiate burrow and time spent burrowing.

### Soil Type Choice Larval Pupation Experiment

To test the soil type that *H. zea* preferred for pupation, we randomly released and spread the 30 larvae across a plastic box (40 cm × 28 cm × 16 cm) (Model 1644LAB8, White, Sterilite, Townsend, MA, USA) equally divided into 2 parts by a piece of plastic (Fig. 1). The design of our soil type choice larval pupation experiment was based on *Spodoptera frugiperda*, also a noctuid species, research comparing pupation in sandy and sandy loam soils (Shi et al. 2021). The soil types used in our study were the same as those used in (Dillard et al. 2023a), who found that *H. zea* burrows averaged around 3 and 5 cm in depth in clay and sandy soil, respectively. Therefore, we added soils to the boxes until reaching 7 cm of height, to allow larvae to burrow at depths similar to field conditions. We set the soil moisture of both soils at 25%, adding water by weight; we kept the boxes at a temperature of 24 °C under ambient light, and we randomly released larvae within boxes. We performed the experiment once in September and once in October 2022. During both experiments, we had 3 replications of each soil type. We released 180 total larvae in this experiment. We counted pupae once all the insects burrowed or died. Pupal depth (live pupae on the soil surface were recorded as 0 cm deep), number of live pupae, and dead insects (pupae and prepupae larvae) were recorded. We classified insects if the larva was not moving and dark-colored or if the pupa was deformed, dark-colored, not moving or did not emerge.

### Soil Moisture Choice Experiment

We divided plastic boxes (Model 1644LAB8) into 4 equal sections with additional plastic. We used hot glue to fill up the gaps between sections and to prevent water permeation. We filled the sections with sandy soil and set moisture levels of 5%, 25%, 50%, and 80% calculated by weight (Fig. 1). As before, we randomly released and spread 30 larvae across each of the boxes. We released a total of 120 larvae in this experiment. In addition, we set a webcam camera (Model 960-000764, Logitech C920, Newark, CA, USA) above the boxes to track the number of deaths and the time larvae took to burrow. We repeated the experiment 4 times (4 boxes) at a constant temperature of 26 °C for replication; to allow the camera to record behavior, we illuminated the experiment with overhead fluorescent lights. We counted pupae once all the insects burrowed or died. We recorded pupae on the soil surface and dead pupae as in the previous experiment. Some larvae pupated on the surface, while some did not pupate or burrow in 36 h of video recording. These were not included in the analysis.

### Soil Moisture No-choice Experiment Pupation and Adult Emergence

We filled plastic cups (Model NCUJ0DS, Chinet Crystal, DeSoto, KS, USA) with sandy soil to 7-cm deep and moisture levels of 0%, 5%, 25%, 50%, and 80% by weight (Fig. 1). We added 1 prepupae larvae *H. zea* to each individual cup. We covered the cups with transparent plastic wrap with small holes to allow airflow but also to avoid significant water evaporation and to allow us to check the presence of adult moths after their emergence. We used a growth chamber at 27 °C and D:L 12:12 for the experiment. We evaluated the number of dead prepupae larvae, prepupae larvae that burrowed, and live and dead pupae on the soil surface, also the total number of adults that emerged daily. Nine days after the first adult moth emerged, all adults had completed emergence. At this time we destructively sampled the soil and counted the number of dead pupae under the soil. We arranged the experiment as a randomized complete block design. We released 135 total larvae in this experiment. We performed the experiment twice, the first one in March and the second one in April/May of 2023 to achieve 2 replications. In the first experiment, we randomly assigned 1 cup of each moisture level in a plastic tray (block) with 10 replications for each treatment, while we used 17 replications in the second experiment.

### Soil Type and Moisture No-choice Adult Emergence Experiment

We conducted a no-choice greenhouse experiment with the same 2 soil types in buckets (Model T808128-2, Berry Plastics) in a 2 × 2 completely randomized factorial design. One factor was soil type, and the other was moisture level. We added 12 laboratory-reared *H. zea* larvae per bucket. In the first treatment, we started both soils at a 5% moisture level set by weight. Seven days after adding larvae, we increased the moisture level in half of the buckets to 50% by slowly pouring water with a watering can so the soil was not disturbed. In the second treatment, both soils were maintained at a 25% moisture level throughout the experiment; we chose this moisture level because *S. frugiperda*, another noctuid, prefers to burrow and pupate in soil between 25% and 50% moisture (Shi et al. 2021). We replicated each treatment 10 times. We released 480 total larvae in this experiment. We sealed the buckets with lids containing small holes to prevent loss of water through evaporation and to allow airflow. We recorded the number of larvae that burrowed after 24 and 48 h and moth emergence daily until emergence ceased as described previously.

### Data Analysis

We transformed data from all experiments before analysis, if needed, to satisfy model assumptions. We also used Tukey’s HSD for mean separations in all analyses. For all analyses, we used individual general linear mixed models ANOVAs (PROC MIXED; SAS 2013).

For the soil type choice larval pupation experiment, we considered the box as the level of replication and ran an analysis of the data from all experiments together. Dependent variables included pupal depth, number of live pupae, and number of dead pupae. Soil type was the fixed factor. Random factors included replication and the interaction of replication and experimental run.

For the soil moisture choice experiment, we considered the number of live pupae remaining on the soil surface, the number of dead pupae remaining on the soil surface, the number of pupae that burrowed and were alive, pupae that were dead, live pupal depth, and dead pupal depth as dependent variables in separate analyses. Soil moisture level was the fixed factor, and the random factors were replication and the interaction of replication and soil moisture level.

For the soil moisture no-choice experiment pupation and adult emergence, we considered the number of pupae on the surface that were alive, the number that burrowed and were alive, and the total number of pupae that were dead (both in the burrow and on the surface; combined since so few were dead on the surface) dependent variables in separate analyses. The soil moisture level was the fixed factor, and replication nested within the experiment and the interaction of soil moisture level and replication nested within the experiment were the random factors. We analyzed adult emergence over time using a repeated measures mixed model ANOVA. The dependent variables were soil moisture level, emergence date, and their interaction. We used the same random factors as the previously described models. Finally, we specified the covariance of the model as compound symmetry since our sampling points were evenly spaced.

For the soil type and moisture no-choice adult emergence experiment, we considered the percentage of pupae that burrowed and the percentage of pupae that died in separate analyses. Fixed factors were soil moisture, soil type, and their interaction. Random factors were replication and the interaction of replication, soil moisture, and soil type. We analyzed adult emergence over time using a repeated measures mixed model ANOVA. We used 7 days of emergency data for this analysis. Because few adults emerged on some days, we analyzed the cumulative number of adults that emerged from an individual bucket over time. The dependent variables were soil moisture level, soil type, emergence date, and their interactions. We used the same random factor as the previously described models. Finally, we specified the covariance of the model as AR (1) since our sampling points were evenly spaced. We looked at significant interactions using the SLICE function in SAS to hold variation by date or soil type constant.

Finally, for the soil type no-choice larval behavior prior to the pupation experiment, we included the dependent variables of time from release to initiation of burrowing, time from initiating burrowing to finish burrowing, the total time to pupation (time since the insect was put in the box, finish burrowing and pupate), pupal depth, and total area of soil that the larvae burrowed in separate analyses. Soil type was the fixed factor, and replication was the random factor.

## Results

### Soil Type No-choice Larval Behavior Prior to Pupation Experiment

The average total tunnel area was different between soil types (*F* = 4.56; *df* = 1,74; *P* = 0.036). Larvae burrowed larger tunnels in the sandy soil (7.67 ± 1.34 cm^2^) compared to the clay soil (4.18 ± 0.55 cm^2^). The time for larvae to initiate burrowing was not different between soil types (*F* = 0.36; *df* = 1,4; *P* = 0.5779). *Helicoverpa zea* initiated burrowing following an average of 431.26 ± 91.09 min of questing time in clay soil compared to an average of 583.61 ± 149.06 min of questing time in sandy soil following larval release. There were no differences for time spent burrowing (1,585.61 ± 255 min in clay and 657.20 ± 129.55 min in sand) or time to reach the pupal stage (59.74 ± 5.36 in clay and 54.23 ± 5.92 min in sand). Finally, we did not find differences in pupal depth between the 2 soils (3.85 ± 0.45 cm in clay and 5.75 ± 0.67 cm for sand).

### Soil Type Choice Larval Pupation Experiment

*Helicoverpa zea* prepupae larvae preferred to pupate more often in sandy soils (81%) compared to clay soils (19%) (*F* = 21.23; *df* = 1,10; *P* < 0.001). However, the mortality of pupae and prepupae larvae was not different between soil types (*F* = 1.47; *df* = 1,10; *P* = 0.2535). An average of 3.5 ± 0.84 prepupae and pupae died in clay and 2.1 ± 0.70 in sand. Finally, pupal depth was not different between soil types (*F* = 0.04; *df* = 1,9; *P* = 0.8504).

### Soil Moisture Choice Experiment

The prepupae took an average of 427.89 ± 35.86 min to burrow (*n* = 64). We did not observe any significant difference among the 4 soil moistures regarding the number of pupae on the soil surface (*F* = 1.93; *df* = 3,9; *P* = 0.1949), number of pupae under the soil (*F* = 0.35; *df* = 3,12; *P* = 0.7904), dead pupae on the soil surface (*F* = 1.90; *df* = 3,9; *P* = 0.1996), or dead pupae in the soil (*F* = 1.22; *df* = 3,12; *P* = 0.3434). Finally, we did not see significant differences in pupal depth (*F* = 0.12; *df* = 3,9; *P* = 0.9483) among soil with 5%, 25%, 50%, and 80% soil moisture levels.

### Soil Moisture No-choice Experiment Pupation and Adult Emergence

**Fig. 2.**
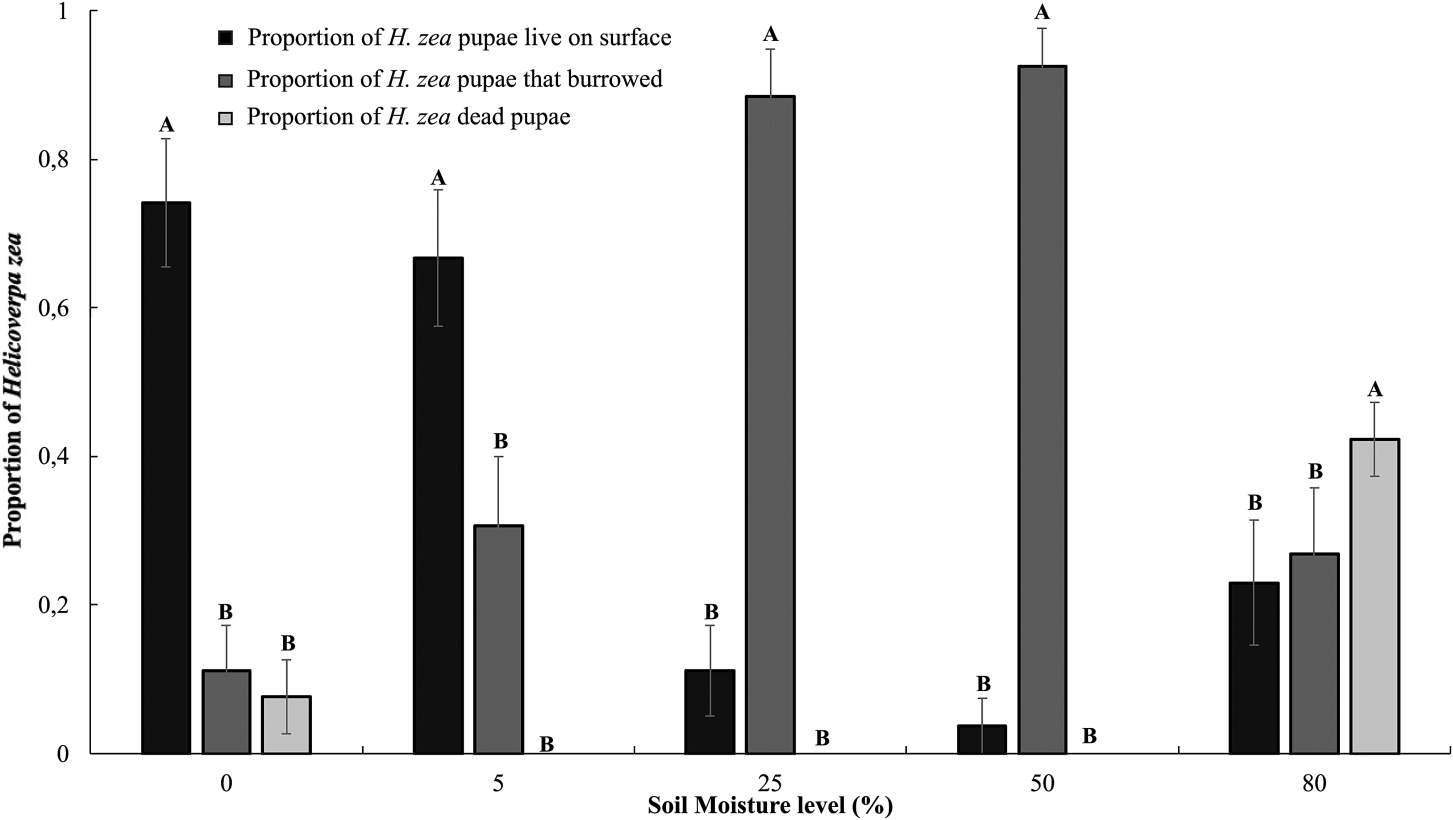
Proportion of live *H. zea* pupae that burrowed and lived or died across different soil moisture levels (0%, 5%, 25%, 50%, and 80%). Note that proportions do not sum to 1 in individual moisture levels because we also counted the number of dead larvae on the soil surface, a factor which was not significantly different across moisture levels. The letters indicate significant differences using the Tukey–Kramer test, *P* < 0.05.

We found that the number of live pupae on the surface was significantly different among moisture levels (Table 3). The proportion of live pupae on the surface was higher in sandy soil with 0% and 5% moisture when compared to soil with intermediate moisture levels of 25%, 50%, and 80% moisture (Fig. 2). The proportion of pupae in the soil was different across moisture levels (Table 3). There were more pupae under the soil with intermediate moisture levels of 25% and 50% compared to the soil at 0%, 5%, and 80% moisture levels (Fig. 2). In addition, the proportion of dead pupae (analyzed as a sum of the dead pupae both on the soil and in the soil) was different across moisture levels (Table 3). There was a higher proportion of dead pupae in the 80% soil moisture level compared to the 0%, 5%, 25%, and 50% soil moisture levels (Fig. 2).

**Table 3.** ANOVA results for the no-choice experiments. *P* < 0.05 *, *P* < 0.01 **, *P* < 0.001***

Experiment	Dependent variables	Independent variables	*df*	*F*	*P*-value
Soil moisture no-choice experiment pupation and adult emergence	Proportion of *H. zea* live pupae on surface	Soil moisture	4, 50.3	21.96	<0.001***
	Proportion of *H. zea* pupae that burrowed		4, 47.6	42.08	<0.001***
	Proportion of *H. zea* dead on the surface or dead that burrowed (total)		4, 103	13.90	<0.001***
	Proportion of adult *H. zea* that emerged	Soil moisture	4, 64.9	9.55	<0.001***
		Date	4, 600	25.15	<0.001***
		Soil moisture * date	16, 600	1.83	0.024*
Soil type and moisture no-choice adult emergence experiment	Total number of *H. zea* larvae that burrowed	Soil moisture	1, 36	8.02	0.007*
		Soil type	1, 36	1.96	0.170
		Soil moisture * soil type	1, 36	2.31	0.137
	Total number of *H. zea* adults that emerged over time	Soil moisture	1, 46.8	0.93	0.338
		Soil type	1, 46.8	2.92	0.094
		Soil moisture * soil type	1, 46.8	0.00	0.974
		Date	6, 218	90.07	<0.001***
		Soil moisture * date	6, 218	1.62	0.143
		Soil type * date	6, 218	7.24	<0.001***
		Soil moisture * soil type * date	6, 218	0.22	0.970

Finally, for the emergence rate, there was an interaction between date and soil moisture level (Table 3). When we held variation among dates constant, emergence among soil moisture levels was significant in only 1 out of 5 sample dates (Table 4). In the second sampling date, the number of adults that emerged was higher in soil with 50% moisture compared to the soil with 0% and 80% moisture levels (Table 4).

**Table 4.** Average number of *H. zea* adults among sampling dates (1, 2, 3, 4, and 5) that emerged across different soil moisture levels (0%, 5%, 25%, 50%, and 80%). The estimate (± SE) within each emergence rate followed by the same letter is not significantly different using the Tukey–Kramer test, *P* < 0.05. Data was not normal, and variances were not heterogeneous

Soil moisture (%)	Date	Proportion of *H. zea* adults that emerged
0	1	0.148 ± 0.069 ns
5		0.111 ± 00.061 ns
25		0.259 ± 00.085 ns
50		0.037 ± 00.061 ns
80		0.000 ± 00.000 ns
0	2	0.296 ± 00.089 b
5		0.444 ± 00.097 ab
25		0.370 ± 00.094 ab
50		0.629 ± 00.094 a
80		0.222 ± 00.081 b
0	3	0.222 ± 00.081 ns
5		0.296 ± 00.089 ns
25		0.185 ± 00.076 ns
50		0.148 ± 00.069 ns
80		0.074 ± 00.051 ns
0	4	0.111 ± 00.061 ns
5		0.037 ± 00.037 ns
25		0.111 ± 00.061 ns
50		0.111 ± 00.061 ns
80		0.000 ± 00.000 ns
0	5	0.000 ± 00.000 ns
5		0.000 ± 00.000 ns
25		0.000 ± 00.000 ns
50		0.000 ± 00.000 ns
80		0.037 ± 00.037 ns

### Soil Type and Moisture No-choice Adult Emergence Experiment

**Fig. 3.**
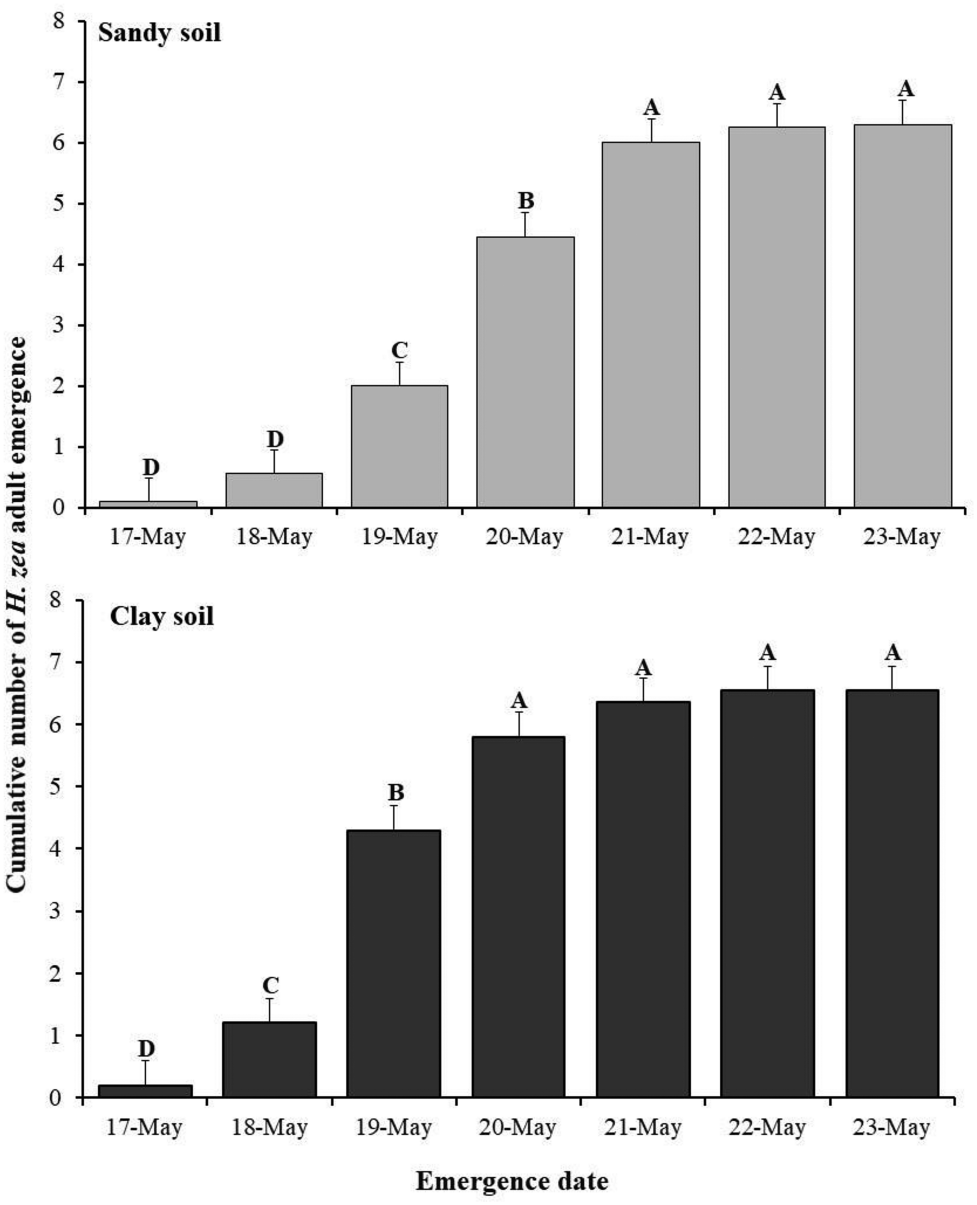
Emergence rate of *H. zea* in sand and clay soils. Soil type and moisture no-choice adult emergence experiment. The letters indicate significant differences using the Tukey–Kramer test, *P* < 0.05.

The number of prepupae larvae that burrowed was different between soil moisture but not between soil type or the interaction (Table 3). There were more prepupae that burrowed in soils that were maintained at 25% moisture (83.3 ± 2.56 larvae) compared to soils that started at 5% moisture and were brought to 50% moisture (68.75 ± 4.63 larvae). We did not find a significant effect on adult emergence for soil moisture level, soil type, or their interaction (Table 3). However, we found a significant effect on adult emergence over time for the interaction of soil type and emergence date (Table 3). Adults emerged at significantly equal frequencies from both soil types across all sampling days (Fig. 3), except on day 3 of emergence. More adults emerged from clay compared to sand on this day (Fig. 3).

## Discussion

In our experiments, *H. zea* adult emergence was not affected by moisture and was only slightly affected by soil type. In contrast, both the soil type and moisture levels had an impact on the burrowing behavior of *H. zea* prepupae larvae and their pupation success. Larvae chose to burrow more often in sand compared to clay; they also burrowed tunnels with a larger area in sand compared to clay. This aligns with previous experiments using the same soil as our experiments, where *H. zea* burrowed deeper and survived better in sand compared to clay (Dillard et al. 2023a). Additionally, in 2 of our experiments (soil moisture no-choice pupation and adult emergence; soil type and moisture no-choice adult emergence), prepupae larvae burrowed more often in intermediate moisture levels and burrowed less frequently in very dry and very wet soil. However, when given a choice (soil moisture choice experiment), *H. zea* did not prefer to burrow in sandy soil with a specific moisture level; we note that this could be an artifact of our choice arena.

Although we did not test the impact of soil texture directly, it was very different between soil types and might be the reason why we observed differences in our results. In soil, the clay fraction is composed of particles smaller than 2 µm, the silt fraction is composed of particles larger than 2 µm, but smaller than 50 µm, and the sand fraction is composed of particles between 50 and 2 µm (Marshall et al. 1996). Therefore, the sandy soil had a coarser texture, while the clay soil had a finer texture. The impact of soil texture on the pupation success of insect species that pupate in the soil is not well-known. However, our choice experiments suggest that fine soil might impede the ability of our focal species, *H. zea*, to burrow prior to pupation. We note that *H. zea* preferred to burrow more often and had higher pupal survival in the sandy soil compared to the clay soil. In a related choice experiment with 4 different soils, fewer *Heortia vitessoides* (Lepidoptera: Crambidae) larvae burrowed and pupated in a silt loam soil (45.4% sand, 52.8% silt, and 1.8% clay), at 20% saturation compared to sand, defined as sandy Loam A (78.6% sand, 10.4% silt, and 11% clay) and sandy Loam B (71.4% sand, 16.6% silt, and 7.63% clay) (Wen et al. 2016). The silt loam soil in this study had a similar amount of sand and silt as the clay soil in our study. Furthermore, the silt loam soil in their study was drier compared to other soils due to differences in water regimes for this soil type, hypothetically making it harder for the insects to burrow compared to the other soils (Wen et al. 2016). However, there are few studies on soil type on the development and survival of lepidopteran prepupae larvae that burrow into the soil and this hypothesis should be tested further.

Our study marks the first examination of the burrowing behavior of *H. zea* larvae relative to soil type and moisture. Prior research has primarily concentrated on the depth at which *H. zea* pupae are found, noting that they tend to be closer to the surface in clay soils as opposed to sandy soils (Phillips and Barber 1929). However, other studies found mixed results regarding the influence of soil type on larvae pupation. Some evaluated the effects of different soils with similar texture compositions (Roach and Hopkins 1979), while others showed that soils with different texture compositions may impede the burrowing of prepupae. This is because dry clay soils form a crust that interferes with prepupae burrowing, a phenomenon that was not observed for sandy loam soils (Landis et al. 1987). We note that we used the same soils as Dillard et al. (2023a, b), who did see differences in burrow depth and survival among soil types. Although we did not find a similar difference in depth between the soil types, we used a much smaller sample size. Hence, our ability to parse differences in depth was likely hampered by a lower power of detection. However, we showed that prepupae behavior differs between soil types with different compositions because *H. zea* prepupae created larger burrows in coarser soil compared to fine-textured soils in our studies. This supports previous results of experiments performed with soils using more extreme composition differences.

We observed that *H. zea* burrowing behavior was similar to that described for *Spodoptera exigua* (Zheng et al. 2011). Larvae of both species use their mandibles to burrow by moving the soil around the body and secreting silk to build the pupal chamber. The disparity we noticed in the tunnel area and depth of *H. zea* between different soil types may be attributed to the insect’s limited ability to use its mandibles to manipulate silt and clay particles, which are more abundant in clay soil. In addition, the time larvae spent burrowing trended higher in clay compared to sandy soil, although this result was not significant. If our hypothesis is correct, the energetic requirements could be different to burrow into these soil types. Nonetheless, since a previous study demonstrated that burrows are shallower in clay soils compared to sandy soils (Dillard et al. 2023a), there may be a set energetic expenditure that prepupae are willing to spend before forming a pupal chamber. However, the lack of significance in our study could have been due to a low sample size and high variation among larvae. Further studies could focus on this hypothesis to explain why they construct shallower pupal chambers in clay soil compared to sandy soil and why they exhibit a preference for pupation in sandy soils.

Our experiment that increased the water content in 2 different soil types had no effect on the emergence of adult *H. zea*. We observed only 1 significant interaction, which was between the soil type on a single date of sampling, but not for moisture (Table 3—soil type and moisture no-choice adult emergence experiment). Apart from the sampling date, no other main effects were significant (Table 3). Our findings align with a previous study that used a single soil type, both dry and saturated, and found that moisture level did not affect the emergence of adult *H. zea* (Dillard et al. 2023b). However, a separate study demonstrated that emergence of adult *S. frugiperda* could be influenced by increasing soil moisture (He et al. 2021). Nevertheless, similar to Shi et al. (2021), there is uncertainty regarding the extent of this effect due to discrepancies among figures, legends, and axes (Fig. 8C and D in He et al. 2021). While more work investigating the impact of moisture on adult emergence is warranted, we did not see a strong influence in our studies.

However, we can confidently conclude that moisture does influence the burrowing behavior of prepupae larvae, because we observed more larvae burrowing when the soil had an intermediate moisture level. This supports the hypothesis that moisture plays a significant role in the pupation behavior of *H. zea* in the soil (Dillard et al. 2023b). In addition, moisture also plays an important role in the burrowing behavior of other species of noctuid. Prepupae of *S. frugiperda* preferred to burrow in intermediate moisture levels (Shi et al. 2021). Furthermore, *H. armigera* and *H. punctigera* pupate is shallower in moisture compared to dry clay soil (Murray and Zalucki 1990). Soil moisture drives the behavior of prepupae, and our results support the evidence that the performance of *H. zea* prepupae pupation is favored by this factor.

When *H. zea* prepupae larvae were given a choice in our experiment, they pupated with equal frequencies across all 4 soil moisture levels; however, when no choice was given, these larvae burrowed more frequently in soil with intermediate moisture levels. The frequency of pupation in soils with different moisture levels has been observed for lepidopteran species in no-choice experiments. In most of these studies, different soil types under dry or wet conditions did not affect the frequency of prepupae entering the soil (Roach and Hopkins 1979, Murray and Zalucki 1990). Similarly, our soil moisture choice experiment showed no influence of soil moisture on *H. zea* prepupae preference for pupation. However, given the results with other lepidopterans (Wen et al. 2016, Shi et al. 2021), our findings might be an artifact of our arena, which contained many individuals in a small area (30 larvae in 1120 cm^2^). For example, in the boxes, individual larvae confronted and evaded other conspecifics—often several times. In some cases, larvae initiated burrowing and were confronted by other wandering larvae; as a result, both larvae would then look for a new place to burrow. We also noticed that larvae only cannibalized other larvae when they were immobile. Conspecifics that were immobile were usually late prepupae or pupae. Perhaps late instar larvae already have sufficient energy to pupate and will only cannibalize conspecifics when the risk is low, as larvae will cannibalize other larvae in a small cage (60 ml tin can, 6 cm diameter, and 2 cm height) even when they have enough energy to complete development (Barber. 1936). Likely, this was another artifact of our study, however, as cannibalism is positively density-dependent (Stinner et al. 1977). Alternatively, there could actually be a difference among lepidopteran species. Future studies could elucidate the preference of *H. zea* pupation, evaluating the choice of individual prepupae in arenas with different moisture levels or comparing species in a single study.

One potential reason why larvae burrow more frequently in sandy soils with intermediate soil moisture may be that they require both silk and moisture in the soil to construct tunnels and the pupal chamber. The moisture in the soil may permit the larvae to more easily manipulate soil particles, as they adhere to them with silk and shape the tunnel; in contrast, larvae in dry soils may be less able to tunnel because they cannot do this as easily. In addition, sandy soils contain a higher proportion of large (relatively clay) sand particles and need water to reach an optimum adhesive force (Li et al. 2022). When larvae move sand particles and produce silk, the tunnels and pupal chamber adhere together and take shape. This burrowing behavior, using silk, soil, and moisture, has also been observed in other noctuid species. For example, *S. exigua* uses silk and saliva to construct its pupal chamber in sandy soils (Zheng et al. 2011). Moreover, investigations into *H. zea* pupation in both dry and moist soils has been conducted under controlled conditions, although the soil type was not specified. In a cage experiment, 2 separate cages protected from rain were filled up with soil. In 1 cage, the soil was kept moist through regular applications of water, while in the other, the soil was kept dry. Prepupae larvae burrowed deeper in dry soils and the mortality rate was also 6 times higher compared to those that burrowed in moister soils (Barber 1941). In our test when the larvae did not have a choice to pupate among moisture levels, mortality was also higher in dryer compared to wetter conditions. Therefore, it seems that the level of soil moisture affects pupal mortality in this species. To better understand this phenomenon, additional research is warranted to explore the underlying mechanism, including its potential impact on tunnel and pupal chamber construction.

In conclusion, soil type and moisture affected *H. zea* late instar larvae burrowing behavior. Specifically, we observed that sandy soils with intermediate moisture levels seemed to encourage larvae to create larger tunnels in the soil. Conversely, we observed that the clay soil posed challenges for burrowing. Conducting additional field studies with varying soil types and moisture levels is essential to corroborate this concept. For instance, future research could explore the influence of parasitoids, such as *Icheneumon promissorius* (Carpenter et al. 1994), on larval burrowing behavior. Additionally, there is potential for practical applications, such as recommending specific tillage machinery suited for different soil types when managing overwintering pupae to control population levels in the soil. However, these recommendations would require further research to assess their feasibility and effectiveness. Finally, this information is important for population modeling (Murray and Zalucki 1990, Lawton et al. 2022) and future potential endeavors in area-wide management of this insect.
